# Crystal structure and characterization of magnesium carbonate chloride hepta­hydrate

**DOI:** 10.1107/S2053229620008153

**Published:** 2020-07-08

**Authors:** Christine Rincke, Horst Schmidt, Gernot Buth, Wolfgang Voigt

**Affiliations:** a Institute of Inorganic Chemistry, TU Bergakademie Freiberg, Leipziger Strasse 29, D-09599 Freiberg, Germany; b Institute for Photon Science and Synchrotron Radiation (IPS), Karlsruhe Institute of Technology (KIT), Hermann-von-Helmholtz-Platz 1, D-76344 Eggenstein-Leopoldshafen, Germany

**Keywords:** magnesium, carbonate, chloride, hydrate, synchrotron, twinning, crystal structure

## Abstract

MgCO_3_·MgCl_2_·7H_2_O is the only known neutral magnesium carbonate con­taining chloride ions at ambient conditions. According to the literature, only small and twinned crystals of this double salt could be synthesised in a concentrated solution of MgCl_2_. For the crystal structure solution, single-crystal diffraction was carried out at a synchrotron radiation source. The monoclinic crystal structure (space group *Cc*) exhibits double chains of MgO octa­hedra linked by corners, connected by carbonate units and water mol­ecules. The chloride ions are positioned between these double chains parallel to the (100) plane. Dry MgCO_3_·MgCl_2_·7H_2_O decom­poses in the air to chlorartinite, Mg_2_(OH)Cl(CO_3_)·*n*H_2_O (*n* = 2 or 3). This work includes an extensive char­acterization of the title com­pound by powder X-ray diffraction, thermal analysis, SEM and vibrational spectroscopy.

## Abstract 2

The structure of the stable double salt MgCO_3_·MgCl_2_·7H_2_O, obtained from concentrated solutions of MgCl_2_, is characterized by double chains of MgO octahedra and carbonate ions parallel to (100), interconnected by chloride ions.

## Introduction   

In the context of CO_2_ research, the inter­actions of CO_2_ with salts and brine solutions are of great inter­est. Therefore, the system MgCl_2_–MgCO_3_–H_2_O–CO_2_ has been investigated. The only nonbasic salt containing carbonate and chloride ions is MgCO_3_·MgCl_2_·7H_2_O (Rincke, 2018 ▸).

The formation conditions of MgCO_3_·MgCl_2_·7H_2_O were described for the first time by Gloss (1937 ▸) and Walter-Levy (1937 ▸). It can be synthesized at room temperature by adding MgCO_3_·3H_2_O to a highly concentrated solution of magnesium chloride saturated with CO_2_ (Gloss, 1937 ▸; Schmidt, 1960 ▸).

Within the scope of outbursts of CO_2_ in potash mines, MgCO_3_·MgCl_2_·7H_2_O was discussed as a storage com­pound for CO_2_ in the 1960s (Schmidt, 1960 ▸; Serowy, 1963 ▸; Serowy & Liebmann, 1964 ▸; Schmittler, 1964 ▸; D’Ans, 1967 ▸). This salt forms needle-like crystals, which are only stable in concentrated MgCl_2_ solution (Moshkina & Yaroslavtseva, 1970 ▸). It decom­poses immediately when it is washed with water. When it was stored in air, basic carbonate was formed (Gloss, 1937 ▸).

Schmittler (1964 ▸) concluded from a powder X-ray diffraction (PXRD) pattern of MgCO_3_·MgCl_2_·7H_2_O that its crystal structure exhibits a *C*-centred monoclinic lattice with parameters *a* = 13.27 (0), *b* = 11.30 (8), *c* = 9.22 (7) Å and β = 118.2 (6)°. Due to the low scattering power and the small size of the crystals, a crystal structure analysis of single crystals was not possible until now. Our own investigations should provide a better com­prehension of the synthesis of MgCO_3_·MgCl_2_·7H_2_O and provide a more detailed characterization, including a crystal structure analysis.

## Experimental   

### Synthesis and crystallization   

The synthesis of MgCO_3_·MgCl_2_·7H_2_O is based on the information of Schmidt (1960 ▸). MgO (1 g, Magnesia M2329, p.a.) was added to 200 g of an aqueous solution of MgCl_2_ (5.5 molal, Fluka, ≥98%). The suspension was stirred for 30 min. Afterwards, the undissolved MgO was filtered off. CO_2_ was bubbled through the stirred solution for 24 h at room tem­per­ature. The product was filtered off for further characterization.

### Single-crystal diffraction   

Data were collected on beamline SCD at the KIT Synchrotron Radiation Source using a Stoe IPDS diffrac­tom­eter with monochromated radiation of λ = 0.8000 Å. A crystal of MgCO_3_·MgCl_2_·7H_2_O was recovered from a droplet of its mother liquor and mounted rapidly in the cold (150 K) stream of nitro­gen gas of the diffractometer.

### Powder X-ray diffraction (PXRD)   

PXRD patterns were taken for phase identification with a laboratory Bruker D8 Discover powder diffractometer in Bragg–Brentano set up (Cu *K*α_1_ radiation, Vantec 1 detector). The samples were prepared as flat plates and measured at room temperature.

### Thermal analysis   

The thermal analysis was performed with a TG/DTA 220 instrument from Seiko Instruments (reference substance: Al_2_O_3_, open platinum crucible; argon flow: 300 ml min^−1^; heating rate: 5 K min^−1^, prior period 30 min at 298.15 K in an argon flow).

### Scanning electron microscopy (SEM)   

The SEM images were recorded with a TESCAN Vega 5130 SB instrument (20 kV accelerating voltage). The sample was coated with gold.

### Vibrational spectroscopy   

For the FT–IR spectrum, a Thermo Scientific Nicolet 380 FTIR spectrometer (spectral resolution: 6 cm^−1^, 256 scans per measurement) with KBr blanks was used.

The Raman spectrum was recorded shortly after synthesis with a Bruker RFS100/S FT spectrometer at room temperature (Nd/YAG-laser, wavelength of the laser: 1064 nm).

### Refinement   

Crystal data, data collection and structure refinement details are given in Table 1 ▸. Due to the small crystals and their low scattering power, the crystal structure solution was carried out by single-crystal diffraction at a synchrotron radiation source. The quality of the crystals affected the measured data set with the effect that only reflections to sin θ_max_/λ = 0.56 Å^−1^ could be considered for the structure refinement. The crystal structure was solved by direct methods. The resulting structure solution exhibits a chemically reasonable atomic arrangement, distances, angles and displacement parameters.

H atoms were placed in the positions indexed by difference Fourier maps and their *U*_iso_ values were set at 1.2*U*_eq_(O) using a riding-model approximation.

The crystal exhibits nonmerohedral twinning. The matrix that relates the individual diffraction pattern was determined as (1 0 1.38, 0 −1 0, 0 0 −1). The reflections of both domains were integrated (number of reflections in domain 1: 2829; domain 2: 3505; overlaid: 641; major twin com­ponent fraction: 56.45%).

## Results and discussion   

### Characterization of magnesium carbonate chloride hepta­hydrate   

**Figure 1:**
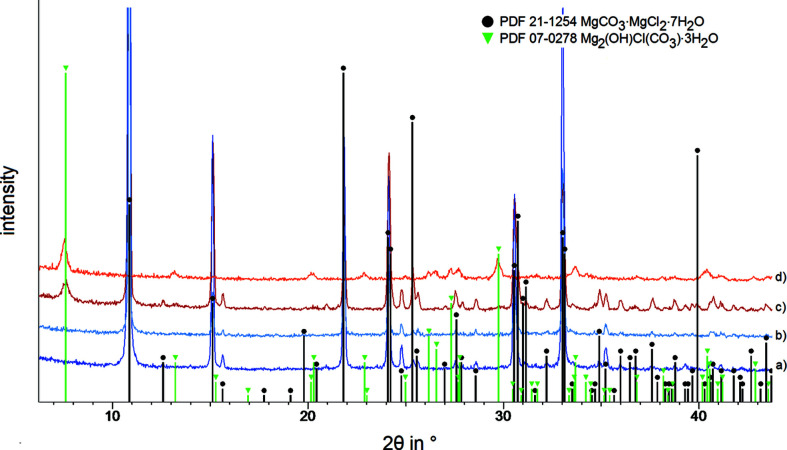
Powder XRD patterns of MgCO_3_·MgCl_2_·7H_2_O under ambient conditions (Cu *K*α_1_ radiation) for (*a*) the unwashed product immediately after the synthesis, (*b*) the unwashed product stored in the air after 19 months, (*c*) the product washed with ethanol after storage in the air for 10 d and (*d*) the product washed with ethanol after storage in the air for 19 months. Reference data: MgCO_3_·MgCl_2_·7H_2_O (PDF 21-1254) and Mg_2_(OH)Cl(CO_3_)·3H_2_O (PDF 07-0278).

The characterization of the unwashed product with PXRD is in accordance with the reference pattern PDF 21-1254 for MgCO_3_·MgCl_2_·7H_2_O (Schmittler, 1964 ▸). The filtered product was stored in a sealed vessel. After 19 months, the powder pattern remained constant, *i.e.* the product did not alter. If the product was washed with ethanol and stored in the air, decom­position to chlorartinite [Mg_2_(OH)Cl(CO_3_)·3H_2_O] begins within a few days (Fig. 1 ▸). This observation confirms the information of Gloss (1937 ▸).

**Figure 2:**
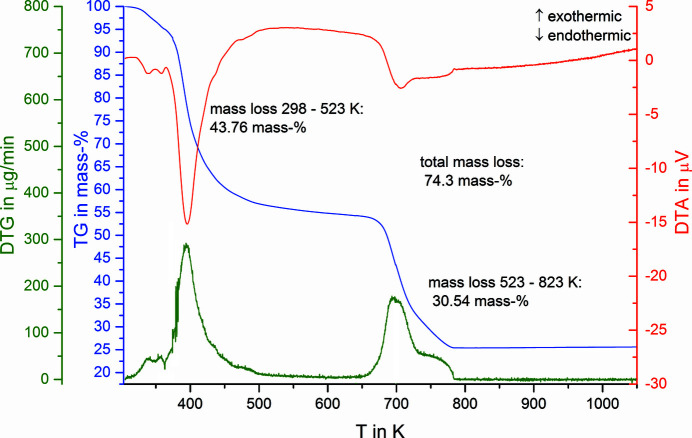
Thermal analysis of MgCO_3_·MgCl_2_·7H_2_O.

The thermal decom­position of MgCO_3_·MgCl_2_·7H_2_O starts as early as the heating begins and shows two main steps (Fig. 2 ▸). H_2_O, CO­_2_ and HCl are evaporated off. This is in accordance with the observation of Serowy & Liebmann (1964 ▸). A precise assignment of the stepwise mass loss is not possible. The characterization of the residue with PXRD at 573 K exhibits the presence of a mixture of basic magnesium carbonates, *i.e.* hydro­magnesite [Mg_5_(CO_3_)_4_(OH)_2_·4H_2_O] and amorphous phases. At 803 K the decom­position is com­plete and only MgO remains in the residue. The observed mass loss of 74.3 (1)% confirms the theoretical mass loss of 73.6%.

**Figure 3:**
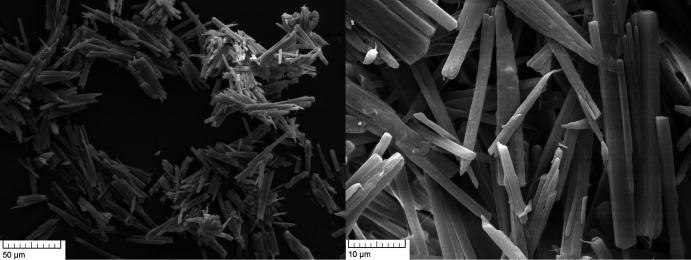
SEM images of MgCO_3_·MgCl_2_·7H_2_O, with the crystals exhibiting twinning or even further inter­growth.

The SEM images of MgCO_3_·MgCl_2_·7H_2_O show thin needles (50 × 5 µm), which are twinned or even more inter­grown (Fig. 3 ▸). Numerous crystallization experiments with the aim of obtaining larger crystals were not successful.

**Figure 4:**
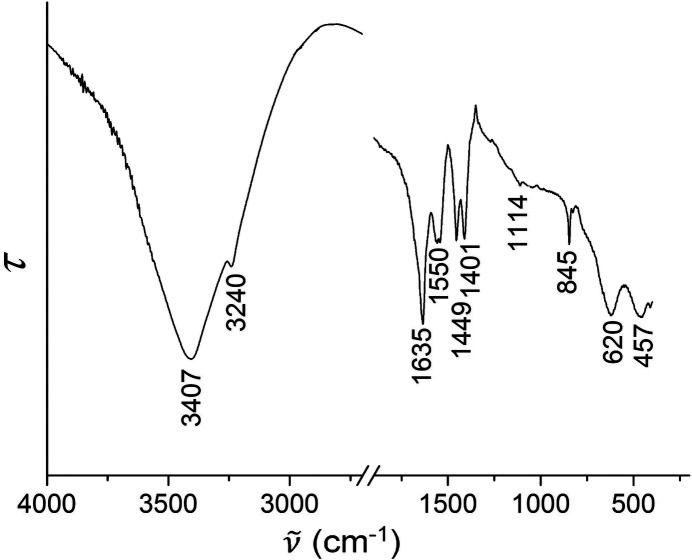
IR spectrum of MgCO_3_·MgCl_2_·7H_2_O under ambient conditions.

**Figure 5:**
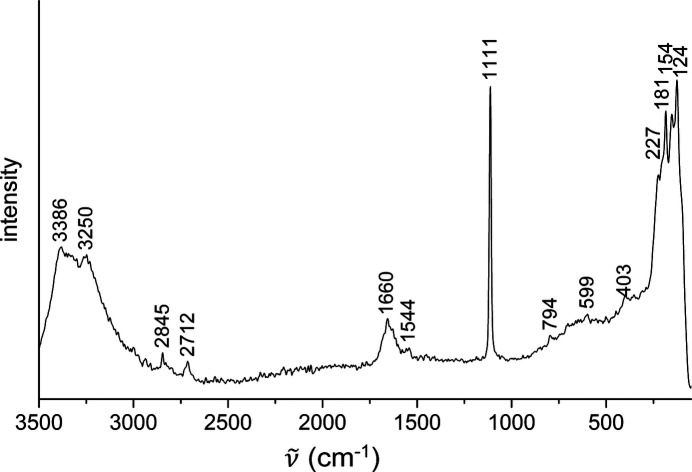
Raman spectrum of MgCO_3_·MgCl_2_·7H_2_O under ambient conditions.

The FT–IR (Fig. 4 ▸) and Raman spectra (Fig. 5 ▸) of MgCO_3_·MgCl_2_·7H_2_O confirm the absence of hydroxide ions in the crystal structure, because there are no bands above 3500 cm^−1^ as in chlorartinite, Mg_2_(OH)Cl(CO_3_)·3H_2_O (Ver­ga­sova *et al.*, 1998 ▸). The assignment of the bands was con­cluded from a com­parison with the vibrational spectra of other neutral magnesium carbonates and chlorartinite (Coleyshaw *et al.*, 2003 ▸; Vergasova *et al.*, 1998 ▸) (Table 2 ▸).

### Crystal structure of magnesium carbonate chloride hepta­hydrate   

**Figure 6:**
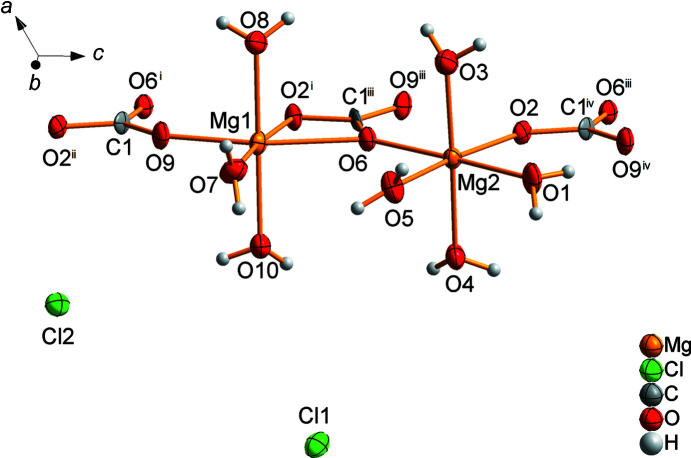
The asymmetric unit and coordination units of MgCO_3_·MgCl_2_·7H_2_O [symmetry codes: (i) *x*, −*y*, *z* − 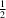; (ii) *x*, *y*, *z* − 1; (iii) *x*, −*y*, *z* + 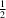; (iv) *x*, *y*, *z* + 1].

**Figure 7:**
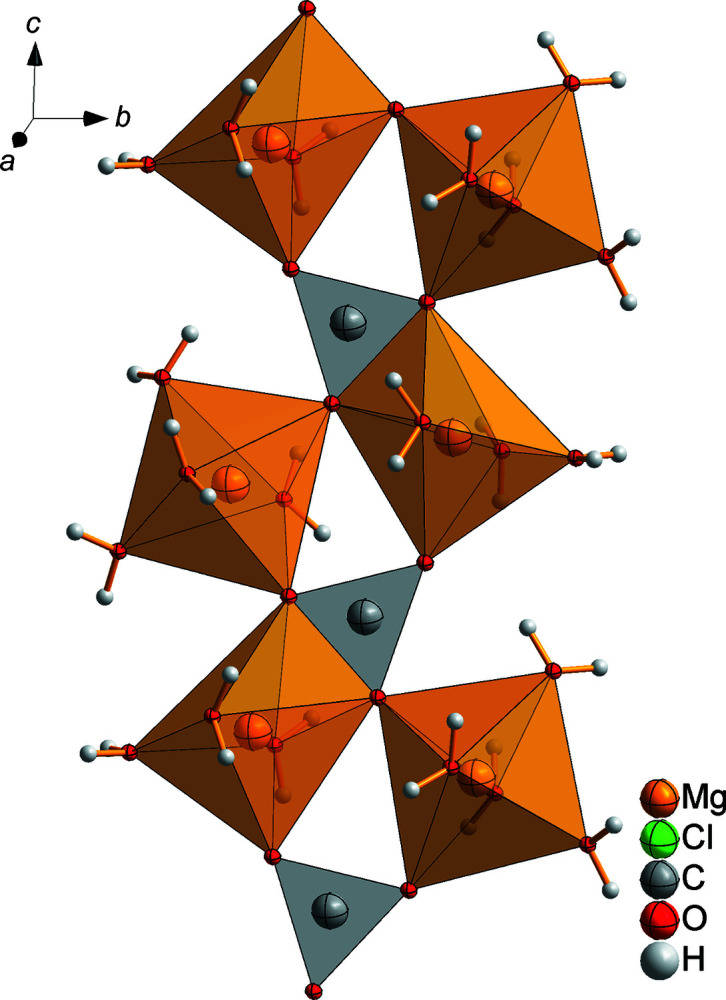
The characteristic structural motif in MgCO_3_·MgCl_2_·7H_2_O, showing the double chain of MgO octa­hedra linked by corners and carbonate units parallel to the (100) plane.

The monoclinic crystal structure of MgCO_3_·MgCl_2_·7H_2_O with the space group *C* *c* and the lattice parameters published by Schmittler (1964 ▸) were confirmed. There are two distinguishable magnesium ions. Mg1 is coordinated by three water mol­ecules and two carbonate anions. One carbonate acts as a monodentate ligand *via* atom O9 and the other as a bidentate ligand *via* atoms O2 and O6. The octa­hedra of Mg2 are formed by four water mol­ecules and two carbonate units which are connected to the magnesium ion in a monodentate manner *via* atoms O2 and O6 (Fig. 6 ▸). The corner-linked Mg–O octa­hedra are arranged in a zigzag manner and together with the car­bon­ate units form double chains parallel to the (100) plane (Fig. 7 ▸).

All the carbonate units are crystallographically equivalent and exhibit a *C*_*s*_ geometry, because they are planar, but the C—O bonds have different lengths. Each carbonate unit is coordinated by three magnesium ions: monodentate to Mg1, bidentate to Mg1^i^ and monodentate to Mg2^ii^ (see Fig. 6 ▸ for symmetry codes). In addition, the carbonate units stabilize the double chains (Fig. 7 ▸).

**Figure 8:**
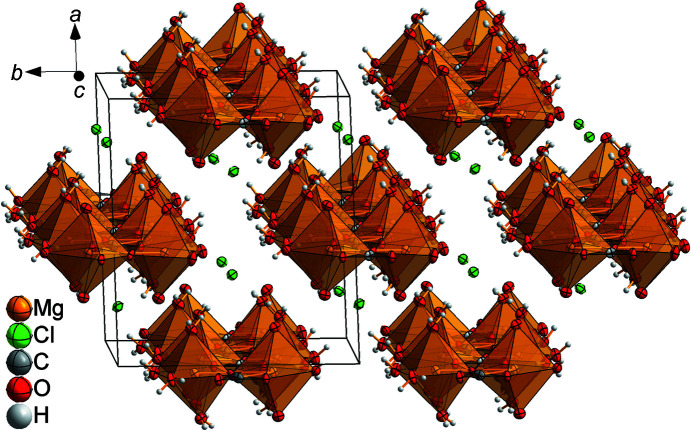
Excerpt of the crystal structure of MgCO_3_·MgCl_2_·7H_2_O, showing the zigzag-like stacking order of the double chains and the chloride ions between them.

**Figure 9:**
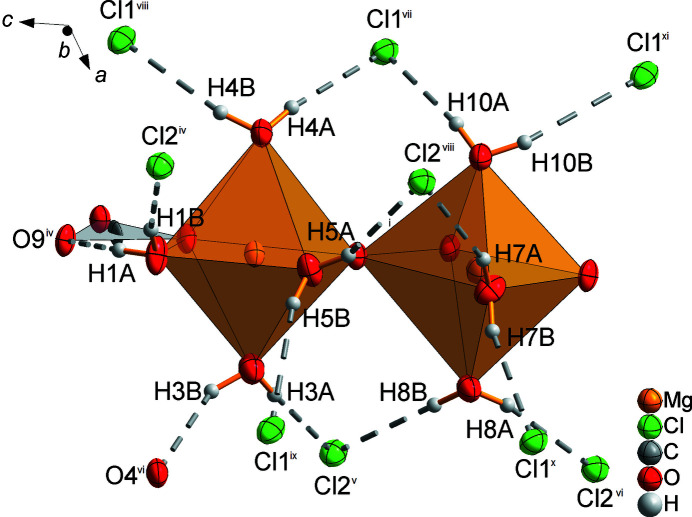
Excerpt of the crystal structure of MgCO_3_·MgCl_2_·7H_2_O, showing the hydrogen-bond inter­actions of the H atoms with chloride ions (dashed lines) [symmetry codes: (iv) *x*, *y*, *z* + 1; (v) *x* + 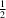, *y* − 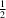, *z* + 1; (vi) *x* + 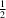, −*y* + 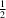, *z* + 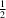; (vii) *x*, *y* − 1, *z*; (viii) *x*, −*y* + 1, *z* + 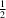; (ix) *x* + 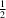, −*y* + 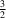, *z* + 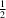; (x) *x* + 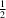, *y* − 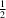, *z*; (xi) *x*, −*y* + 1, *z* − 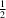].

Between the double chains, which are arranged in a zigzag-like stacking order parallel to the (001) plane, are located the chloride ions Cl1 and Cl2 (Fig. 8 ▸). The positions of atoms H1*A* and H3*B* are fixed by short hydrogen bonds to atoms O9^iv^ and O4^vi^, and the other H atoms by inter­actions with the chloride ions (Table 3 ▸ and Fig. 9 ▸). As a consequence, a three-dimensional network is formed.

The structural motifs of such double chains are similar in MgCO_3_·MgCl_2_·7H_2_O and MgCO_3_·3H_2_O (Giester *et al.*, 2000 ▸), but in contrast to MgCO_3_·3H_2_O in MgCO_3_·MgCl_2_·7H_2_O, only two of three carbonate units and three and four water mol­ecules instead of two water mol­ecules are linked to each Mg atom. Furthermore, no free water mol­ecules are positioned between these double chains in MgCO_3_·MgCl_2_·7H_2_O. The crystal structures of other neutral magnesium carbonates, *e.g.* MgCO_3_·5H_2_O, MgCO_3_·6H_2_O and the chloride-containing magnesium carbonates Mg_2_(OH)Cl(CO_3_)·2H_2_O (chlorartinite) and Mg_2_(OH)Cl(CO_3_)·H_2_O (dehydrated clorarti­nite), do not exhibit such double chains (Liu *et al.*, 1990 ▸; Rincke *et al.*, 2020 ▸; Sugimoto *et al.*, 2006 ▸, 2007 ▸). Therefore, the crystal structure of MgCO_3_·MgCl_2_·7H_2_O is unique.

## Supplementary Material

Crystal structure: contains datablock(s) I. DOI: 10.1107/S2053229620008153/uk3196sup1.cif

Structure factors: contains datablock(s) I. DOI: 10.1107/S2053229620008153/uk3196Isup2.hkl

Supporting information file. DOI: 10.1107/S2053229620008153/uk3196Isup3.cml

CCDC reference: 2010753

## Figures and Tables

**Table 1:** Experimental details

Crystal data	
Chemical formula	MgCO_3_·MgCl_2_·7H_2_O
*M*_r_	305.64
Crystal system, space group	Monoclinic, *C* *c*
Temperature (K)	150
*a*, *b*, *c* (Å)	13.368 (5), 11.262 (5), 9.266 (4)
β (°)	118.83 (3)
*V* (Å^3^)	1222.0 (9)
*Z*	4
Radiation type	Synchrotron, λ = 0.8000 Å
μ (mm^−1^)	0.93
Crystal size (mm)	0.13 × 0.07 × 0.01 × 0.02 (radius)

Data collection	
Diffractometer	Stoe IPDS II
Absorption correction	For a sphere (Coppens, 1970 ▸)
No. of measured, independent and observed [*I* > 2σ(*I*)] reflections	8746, 6975, 5476
*R*_int_	0.0613
θ_max_ (°)	26.7
(sin θ/λ)_max_ (Å^−1^)	0.561

Refinement	
*R*[*F* ^2^ > 2σ(*F* ^2^)], *wR*(*F* ^2^), *S*	0.053, 0.161, 1.12
No. of reflections	4791
No. of parameters	179
No. of restraints	22
H-atom treatment	Only H-atom coordinates refined
Δρ_max_, Δρ_min_ (e Å^−3^)	0.36, −0.43
Absolute structure	Flack *x* determined using 647 quotients [(*I* ^+^) − (*I* ^−^)]/[(*I* ^+^) + (*I* ^−^)] (Parsons *et al.*, 2013 ▸)
Absolute structure parameter	0.43 (13)

Computer programs: *X-AREA* (Stoe & Cie, 2015 ▸), *X-RED* (Stoe & Cie, 2015 ▸), *SHELXS97* (Sheldrick, 2008 ▸), *SHELXL2016* (Sheldrick, 2015 ▸), *DIAMOND* (Brandenburg, 2017 ▸) and *publCIF* (Westrip, 2010 ▸).

**Table 2:** Assignment of the IR and Raman bands of MgCO_3_·MgCl_2_·7H_2_O

IR	Raman	Assignment (Coleyshaw *et al.*, 2003 ▸)
3407, 3240	3386, 3250	ν(OH)_W_
1635	1660	δ(OH)_W_
1550, 1449, 1401	1544	ν_as_(CO)
1114	1111	ν_*s*_(CO)
845	794	γ(CO)
620	599	δ_as_(CO)
457	403, 227, 181, 154, 124	lattice vibrations

Notes: ν = valence vibration, δ = deformation vibration (in the plane), γ = deformation vibration out of the plane, W = water, s = symmetric and as = asymmetric.

**Table 3:** Hydrogen-bond geometry (Å, °)

*D*—H⋯*A*	*D*—H	H⋯*A*	*D*⋯*A*	*D*—H⋯*A*
O1—H1*A*⋯O9^iv^	0.82 (3)	1.94 (6)	2.688 (14)	153 (13)
O1—H1*B*⋯Cl2^iv^	0.82 (3)	2.38 (4)	3.186 (11)	167 (12)
O3—H3*A*⋯Cl2^v^	0.82 (3)	2.32 (3)	3.135 (11)	174 (17)
O3—H3*B*⋯O4^vi^	0.82 (3)	2.10 (11)	2.796 (13)	143 (17)
O4—H4*A*⋯Cl1^vii^	0.82 (3)	2.36 (3)	3.176 (10)	171 (14)
O4—H4*B*⋯Cl1^viii^	0.82 (3)	2.49 (7)	3.251 (10)	155 (13)
O5—H5*A*⋯Cl2^viii^	0.82 (3)	2.45 (7)	3.222 (11)	157 (16)
O5—H5*B*⋯Cl1^ix^	0.81 (3)	2.54 (6)	3.327 (11)	164 (17)
O7—H7*A*⋯Cl2^viii^	0.82 (3)	2.42 (6)	3.212 (11)	163 (16)
O7—H7*B*⋯Cl1^x^	0.82 (3)	2.30 (4)	3.111 (11)	169 (16)
O8—H8*A*⋯Cl2^vi^	0.82 (3)	2.46 (5)	3.254 (10)	163 (11)
O8—H8*B*⋯Cl2^v^	0.82 (3)	2.41 (3)	3.233 (10)	178 (11)
O10—H10*A*⋯Cl1^vii^	0.82 (3)	2.34 (5)	3.146 (10)	166 (15)
O10—H10*B*⋯Cl1^xi^	0.82 (3)	2.67 (7)	3.424 (10)	154 (12)

Symmetry codes: (iv) *x*, *y*, *z* + 1; (v) *x* + 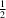, *y* − 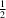, *z* + 1; (vi) *x* + 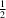, −*y* + 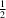, *z* + 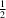; (vii) *x*, *y* − 1, *z*; (viii) *x*, −*y* + 1, *z* + 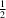; (ix) *x* + 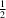, −*y* + 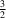, *z* + 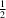; (x) *x* + 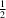, *y* − 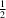, *z*; (xi) *x*, −*y* + 1, *z* − 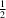.
